# Distinct genomic profile in *h. pylori*‐associated gastric cancer

**DOI:** 10.1002/cam4.3765

**Published:** 2021-03-09

**Authors:** Xiaochen Zhang, Fang Liu, Hua Bao, Ao Wang, Ming Han, Xue Wu, Yanhong Gu, Leizhen Zheng

**Affiliations:** ^1^ Department of Medical Oncology The First Affiliated Hospital College of Medicine Zhejiang University Hangzhou Zhejiang China; ^2^ Department of Radiation Oncology Chinese PLA General Hospital Beijing China; ^3^ Nanjing Geneseeq Technology Inc. Nanjing Jiangsu China; ^4^ Department of Oncology and Cancer Rehabilitation Center The First Affiliated Hospital of Nanjing Medical University Nanjing Jiangsu China; ^5^ Department of Oncology Xinhua Hospital School of Medicine Shanghai Jiao Tong University Shanghai China

**Keywords:** cancer biology, cancer genetics, cancer risk factors, gastric cancer

## Abstract

Gastric cancer is one of the most common and deadly cancer types. Currently, four subtypes have been identified with unique molecular alterations: Epstein–Barr virus (EBV)‐positive, microsatellite instability (MSI), chromosomal instability (CIN), and genomic stable (GS) tumors. Notably, many gastric tumors are associated with the bacterium Helicobacter pylori but the genomic landscape of this subgroup of tumors remains largely unknown. Targeted sequencing covering 425 genes was performed retrospectively on 1703 gastric tumor tissues and matched normal blood samples. Nonsynonymous mutations, copy‐number variation (CNV), and MSI status were called from human DNA reads; nonhuman DNA reads were mapped to NCBI microbial reference genome using Kraken and significant species were identified. Overall, 37 (2.76%) from a total of 1703 samples were EBV‐positive, 200 (11.74%) samples were H. pylori‐positive, and 10 samples were positive for both. Among the rest, 59 (3.46%) samples were MSI, 380 (22.31%) were CIN, and 1017 (59.72%) were GS. Most of the 200 H. pylori‐positive samples tend to be genome stable (85.5%, *p* < 0.001) and microsatellite stable (95%, *p* = 0.04). Compared to 1017 GS tumors, mutations in *AKT3*, *EPAS1*, *MLH1*, and *BKT* and amplifications of *NFE2L2*, *TERC*, *MCL1*, and *TOP1* were significantly enriched in H. pylori‐positive tumors. And compared to EBV‐positive tumors, mutations in *PIK3CA*, *ARID1A*, and *PTEN* were significantly depleted in H. pylori‐positive subtype while *TP53* mutations were enriched. This study characterized the unique genomic landscape of H. pylori‐positive gastric tumors using targeted panel sequencing. The successful identification of DNA reads from infectious agents in tumor samples indicates that deep sequencing is a promising way to uncover characteristics of microbial environment in tumors.

## Graphical abstract

Previous group have found 4 molecular subtypes for gastric adenocarcinoma. We uncovered an additional subtype of H.pylori associated gastric cancer with distinct genomic profile.

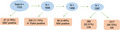

## 1 INTRODUCTION

Gastric cancer is one of the most common and deadly cancer with a 5‐year survival of 30%.^1^, ^2^ Historically East Asia has the highest incidence rate of gastric cancer with China alone accounting for more than half of global deaths.^3^, ^4^, ^5^ Based on histological properties, gastric cancer can be classified into intestinal and diffuse types according to the Lauren Classification.^6^ Alternatively, based on unique molecular alterations detected by next‐generation sequencing (NGS) technology, currently four molecular subtypes have been characterized: Epstein–Barr virus (EBV)‐positive, microsatellite instability (MSI), chromosomal instability (CIN), and genomic stable (GS) tumors.^7^ Besides EBV infection, many gastric tumors are associated with infection of the bacterium Helicobacter pylori (H. pylori) and the frequencies of EBV and H. pylori infection vary depending on geographical locations.^8^, ^9^ H. pylori is a helix‐shaped Gram‐negative bacterium usually found in the stomach.^10^ It is known to cause gastritis and gastric ulcer through chronical inflammation, which may later develop into cancerous tissues.^11^, ^12^ Despite the important association between H. pylori and gastric cancer, the genomic landscape of H. pylori‐positive gastric tumors remains largely unknown. There is some evidence that H. pylori could activate the oncogenic drivers *EGFR* and *Akt* signaling, both oncogenic drivers, in gastric cancer cells.^13^ Others found that the H. pylori virulence factor CagA can inactivate an important tumor suppressor *RUNX3* which may lead to gastric carcinoma.^14^, ^15^ However, these studies were at the gene expression or epigenetic level, and it is still unclear whether and which specific genomic alterations correlate with H. pylori‐positive gastric cancers.

To uncover potential correlations, we retrospectively examined the mutational landscape of 1703 gastric tumor tissues using a hybridization capture‐based NGS panel. Bacterial and viral DNA reads were extracted from both tumor and paired whole blood samples to construct the microbial profile of gastric cancers. By comparing the genomic landscape between two groups of patients with high or low H. pylori levels in their tumor tissues, we identified unique genomic alterations underlying H. pylori‐positive tumors with biological and clinical implications.

## 2 METHODS

### 2.1 Patients

We retrospectively examined data from 1703 primary gastric cancer tissue samples sequenced using a customized targeted sequencing panel. All of these samples were passed in‐house QC procedures including FFPE damage, contamination, and matched normal control tests. All of 1703 samples contained at least one somatic mutation or CNV. Written informed consent was collected from each patient upon sample collection according to the protocols approved by the ethical committee of their respective hospitals. In patients with available clinical data: the male to female ratio was 2:1. There was more than twice the number of patients under age 65 compared to above age 65.

### 2.2 DNA extraction, library preparation, and sequencing

Genomic DNAs from FFPE samples and whole blood control samples were extracted with QIAamp DNA FFPE Tissue Kit and DNeasy Blood and tissue kit (Qiagen), respectively, and quantified by Qubit 3.0 using the dsDNA HS Assay Kit (ThermoFisher Scientific). Library preparations were performed with KAPA Hyper Prep Kit (KAPA Biosystems). A customized panel targeting 425 cancer‐relevant genes was used for hybridization enrichment (Appendix [Link], [Link]). The capture reaction was performed with Dynabeads M‐270 (Life Technologies) and xGen Lockdown hybridization and wash kit (Integrated DNA Technologies) according to manufacturers’ protocols. Captured libraries were on‐beads PCR amplified with Illumina p5 (5’ AAT GAT ACG GCG ACC ACC GA 3’) and p7 primers (5’ CAA GCA GAA GAC GGC ATA CGA GAT 3’) in KAPA HiFi HotStart ReadyMix (KAPA Biosystems), followed by purification using Agencourt AMPure XP beads. Libraries were quantified by qPCR using KAPA Library Quantification kit (KAPA Biosystems). Library fragment size was determined by Bioanalyzer 2100 (Agilent Technologies). The target‐enriched library was then sequenced on HiSeq4000 NGS platforms (Illumina) according to the manufacturer's instructions.

### 2.3 Mutation calling, MSI, TMB, CNV, and CIS calculation

Trimmomatic was used for FASTQ file quality control. Leading/trailing low quality (quality reading below 20) or N bases were removed. Pair‐end reads were then aligned to the human reference genome (hg19) using Burrows‐Wheeler Aligner (BWA) with default parameters. PCR deduplication was performed using Picard V2.9.4 (Broad Institute). Local realignment around indels and base quality score recalibration was performed with the Genome Analysis Toolkit (GATK 3.4.0). Somatic single‐nucleotide variants (SNVs) were identified using MuTect, and small insertions and deletions (indels) were detected using SCALPEL. Final list of mutations was annotated using vcf2maf (available on GitHub). The resulted mutation lists were filtered through an internally collected list of recurrent sequencing errors on the same sequencing platform, which is summarized from the sequencing results of 500 normal samples with a minimum average sequencing depth of 700×. Specifically, if a variant was detected (i.e. ≥3 mutant reads and >1% VAF) in >10% of the normal samples, it was considered a likely artifact and was removed. Mutations that occurred within the repeat masked regions were also removed. In a further filtering step, the mutation was only called out when the VAF is above 1% with a minimum of three mutant reads for hotspot COSMIC mutations (>20 recurrences), or above 2% with a minimum of five mutant reads for other mutations. ANNOVAR ^16^ was used to annotate mutations with variant type, dbSNP ID, clinical significance, and protein impact prediction with SIFT^17^ and PolyPhen.^18^

MSI was estimated based on 52 indel sites in Geneseeq Prime panel. If >40% of 52 sites showed unstable status (compared to the distribution of 500 normal sample pools), the sample was identified as MSI. TMB was counted by summing all base substitutions and indels in the coding region of targeted genes, including synonymous alterations to reduce sampling noise and excluding known driver mutations as they are over‐represented in the panel. Gene CNVs were identified with log2 depth ratio >0.6 for copy number gain and <−0.6 for copy number loss. The average proportion of the genome with aberrant (log2 depth ratio >0.2 or <−0.2) copy number, weighted on each of the 22 autosomal chromosomes, was estimated as the CIS (chromosomal instability score). Assay validations of mutation calling, CNV, MSI, and TMB determination were performed with CLIA/CAP accreditation.

### 2.4 Viral and bacterial reads identification

To identify microbial DNA sequences in samples, reads that mapped to hg19, mitochondrial genomes or bacterial plasmids were removed (NCBI RefSeq database, accessed on July 19, 2018). The remaining reads were mapped to NCBI microbial reference genome databases using k‐mer‐based algorithm with Kraken. Relative abundance at bacteria species and genus level was estimated for each sample by Bracken with recommended parameters.

Since EBV and H. pylori presence was detected using targeted sequencing off‐target reads, only tumors with a high level of EBV or H. pylori reads are detected and classified as EBV or H. pylori‐positive (relatively high abundance). Samples with no EBV or H. pylori reads detected are classified as EBV or H. pylori‐negative (relatively low abundance). The genomic landscapes of two groups of patients with high or low H. pylori levels in their tumor tissues were then compared in this study.

### 2.5 Statistical analysis

Associations between categorical variables were examined using the Chi‐square or Fisher's exact test (only genes with mutations or CNVs in more than 10 samples were included in tests). Association between categorical and numerical variables were compared using Wilcoxon test. A two‐sided p‐value of less than 0.05 was considered statistically significant. P‐values were FDR adjusted for multiple hypothesis testing correction, and adjusted p‐value of less than 0.2 was considered statistically significant. All statistical analyses were performed in R (v.3.3.2).

## 3 RESULTS

### 3.1 Samples

**FIGURE 1:**
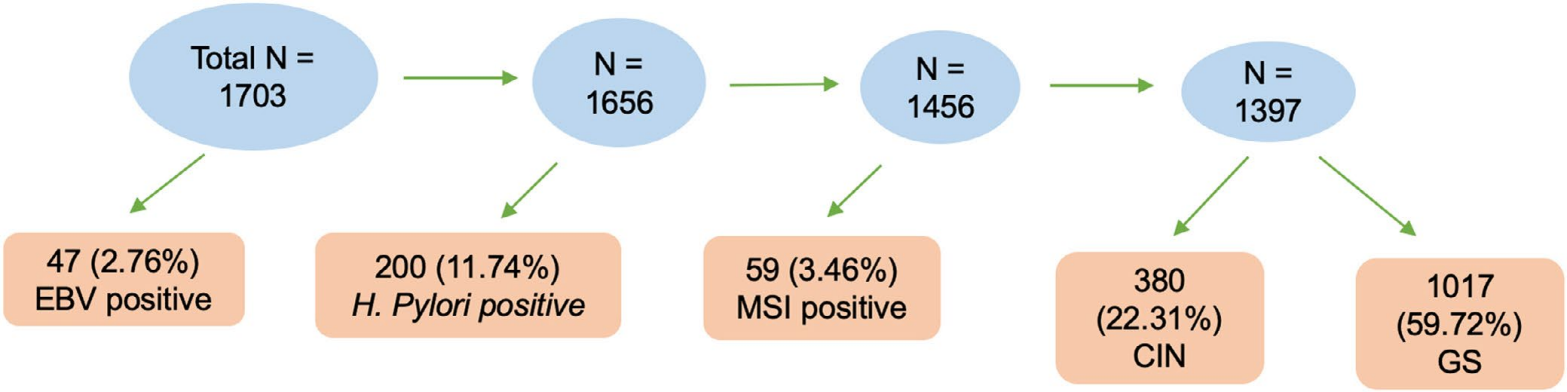
Sample classification results

Sequencing results from 1703 primary gastric cancer tissue samples were analyzed in our study. Following a similar classification framework outlined in the TCGA study^2^, if any EBV sequence was detected in a sample, then, this sample is categorized as EBV‐positive. H. pylori‐positive sample is similarly defined. For EBV and H. pylori‐negative samples, a sample was categorized as MSI if that sample had an MSI score equal or greater than 0.4; a sample was categorized as CIN if that sample had a CIS score equal or greater than 0.25 (top quartile). Samples that did not meet any of the above criteria were categorized as GS. Overall, 47 (2.76%) samples were EBV‐positive and 200 (11.74%) samples were H. pylori‐positive (Figure 1). Additionally, 10 samples were positive for both infections but were counted in the EBV‐positive subtype for all analyses. No significant bacterial or viral reads were found in normal blood. Among the rest, 59 (3.46%) samples were MSI, 380 (22.31%) were CIN, and 1017 (59.72%) were GS.

### 3.2 Genome instability

**FIGURE 2:**
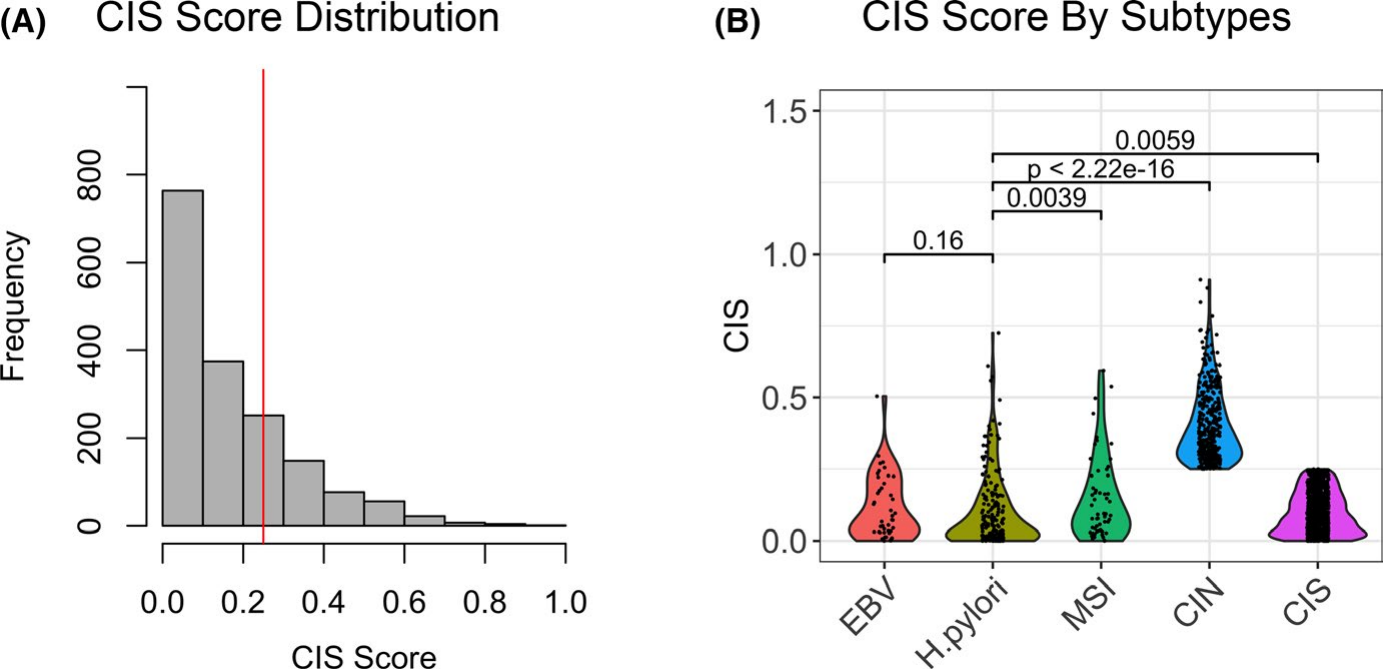
CIS. (A) Distribution of CIS among 1703 tissue samples. The red vertical line denotes top quartile (CIS =0.25). (B) Violin plot of CIS among EBV, H. pylori, MSI, CIN, and GS groups, respectively. There was no difference between EBV and H. pylori groups but the other three groups had significantly higher CIS

The distribution of CIS was right‐skewed with a mean value of 0.12 (range 0–0.91, Figure 2A). The CIN group had the highest CIS value. Among the other four groups, the CIS values between EBV‐positive and H. pylori‐positive samples were not significantly different (median 0.07 vs. 0.06, respectively, *p* = 0.16, Figure 2B), but H. pylori‐positive subtype had lower CIS value than MSI (median 0.06 vs. 0.09, *p* = 0.0039) and GS subtypes (median 0.06 vs. 0.08, *p* = 0.0059).

Patients that were EBV‐positive tend to be younger (median age 54.5 years) while patients with CIN subtype tend to be older (median 61 years). This likely reflects the accumulation of genomic alteration as a function of time.^19^ There were no significant differences in sex ratio among subtypes. MSI subtypes had significantly higher median TMB than the other groups (Table 1). Interestingly, non‐MSI subtypes had similar median TMB and H. pylori‐positive samples had lower MSI score than other non‐MSI subtypes although it was not significant.

**TABLE 1:** Clinical and genomic details among five gastric cancer subtypes

	EBV	H. pylori	MSI	CIN	GS	*p* value
Median Age (yrs)	54.5	58	58	61	57	0.0003
Male ratio	76.6%	71.5%	69.5%	73.2%	65.5%	0.063
Median TMB	7	8	39	7	7	< 0.0001
Median MSI Score	0.0385	0.023	0.6923	0.0385	0.0385	< 0.0001

No significant difference in male ratio among groups. CIN group was the oldest and EBV group was the youngest. MSI group had significantly higher TMB and MSI scores than all other groups.

### 3.3 Mutational signature

**FIGURE 3:**
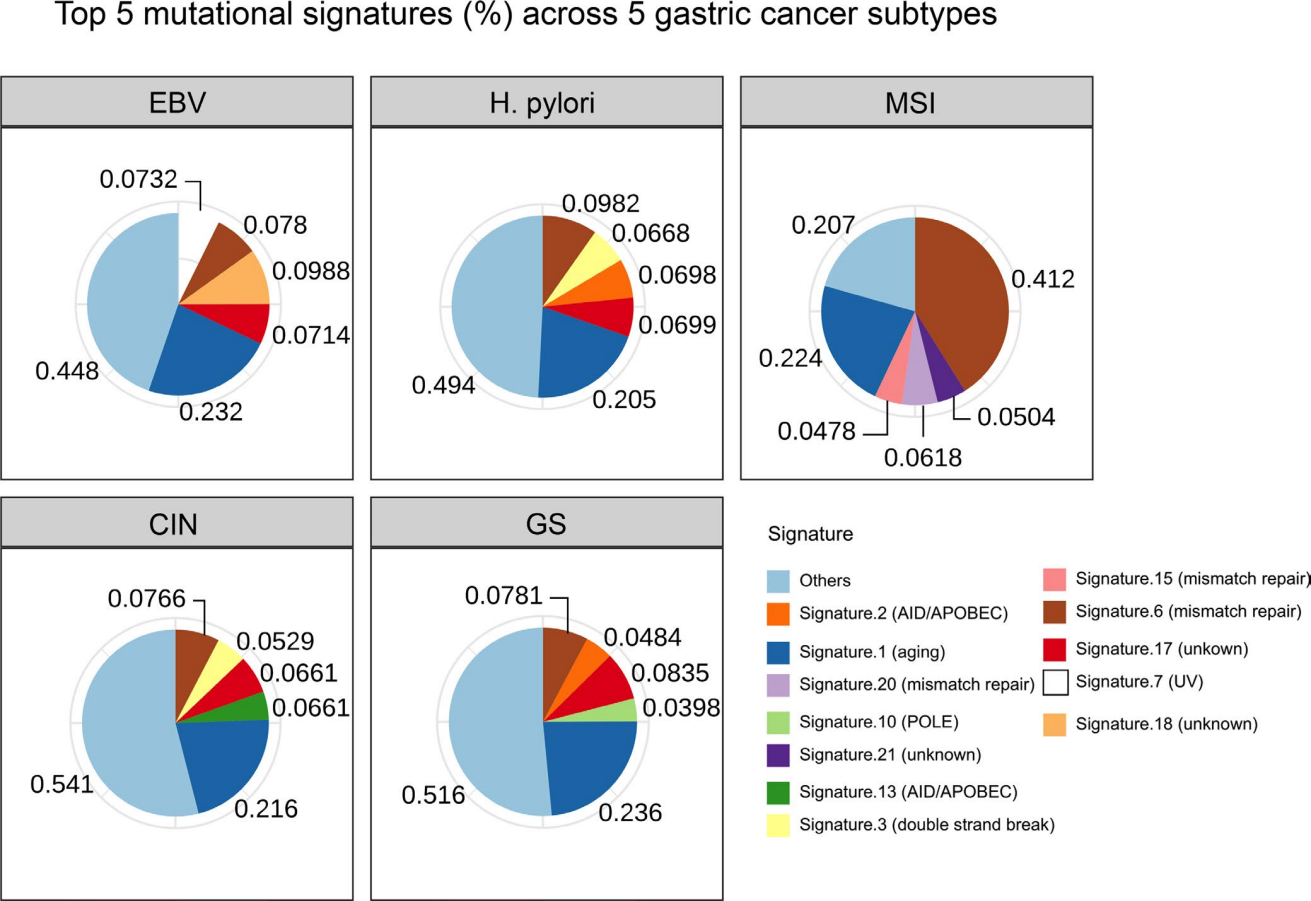
Top five mutational signatures (%) in each subtype. Aging signature was highly prevalent in all groups. Signatures related to mismatch repair, double‐strand break, and APOBEC were also common in H. pylori group. The CIN group was the most similar to H. pylori group

Next, we examined the mutational signature of each sample (Figure 3). Aging signature was highly prevalent in all groups, ranking number 1 in all but MSI subtypes. In MSI tumors, signals were dominated by mismatch‐repair deficiency‐related signatures (52%), which is a known cause of MSI in cancer genome.^20^ Furthermore, signatures related to mismatch repair, double‐strand break, and APOBEC were common in H. pylori group. Notably, CIN subtype had a very similar mutational signature profile compared to H. pylori‐positive subtype. About 5% of signature were attributed to double‐strand break in CIN, which likely contributed to the high CIS score in this subtype. Except for the POLE‐related signature, the GS subtype also had a similar mutational signature profile compared to H. pylori‐positive subtype. Interestingly, EBV‐positive tumors had 7% of signatures related to UV exposure.

### 3.4 Specific mutations and CNVs

**FIGURE 4:**
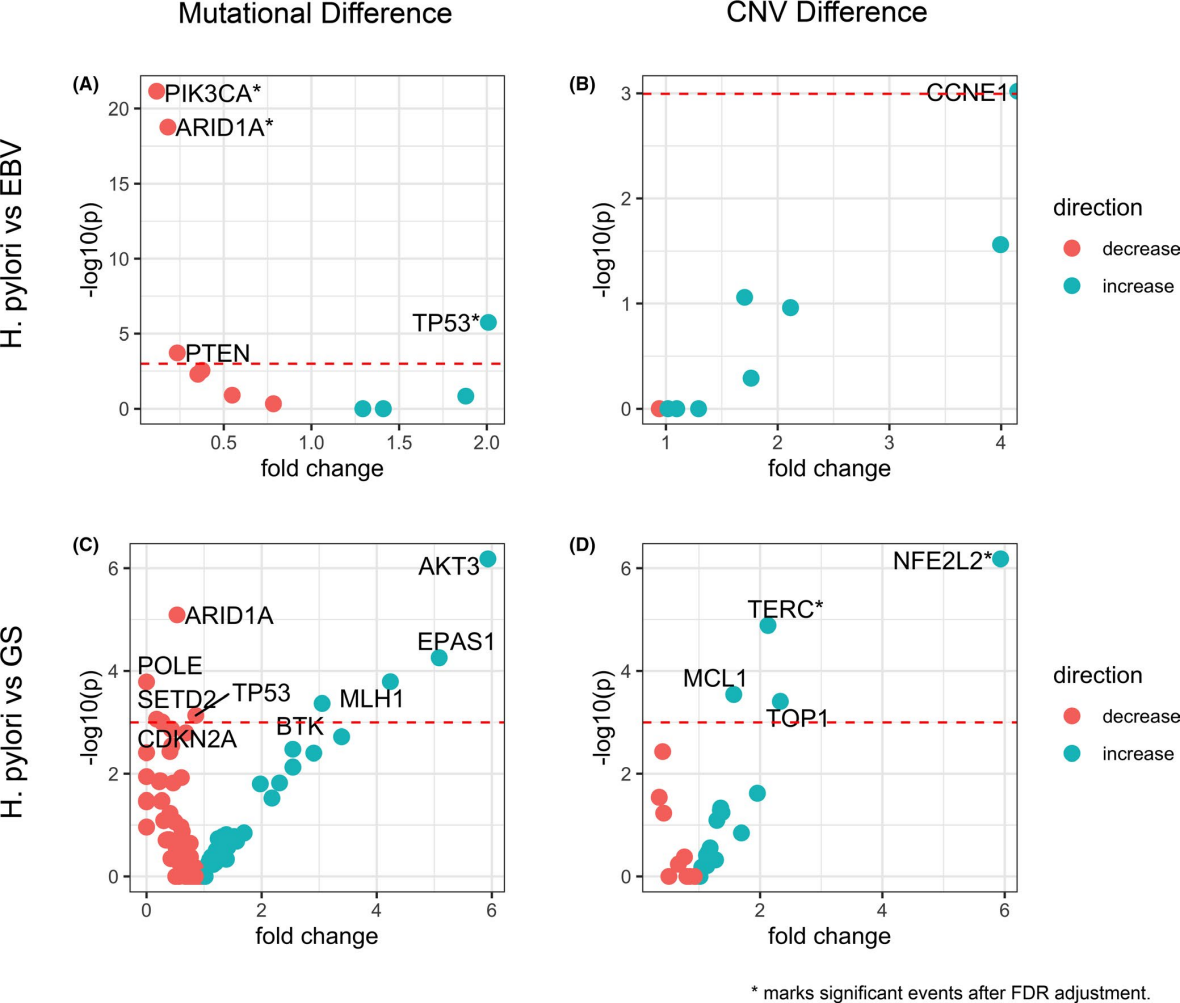
Event enrichment for mutations and CNVs in gastric cancer subtypes. (A) Mutations enriched in H. pylori versus EBV subtypes. Events enriched in H. pylori are blue and those enriched in EBV are red. Significant events are labeled. * marks significant events after FDR adjustment. (B) CNVs enriched in H. pylori versus EBV subtypes. Events enriched in H. pylori are blue and those enriched in EBV are red. The significant event is labeled and is copy number gain. (C) Mutations enriched in H. pylori versus GS subtypes. Events enriched in H. pylori are blue and those enriched in GS are red. Significant events are labeled. (D) CNVs enriched in H. pylori versus GS subtypes. Events enriched in H. pylori are blue and those enriched in GS are red. Significant events are labeled and all are copy number gains. * marks significant events after FDR adjustment

Because EBV and H. pylori‐positive subtypes are both associated with infectious agents, we examined mutations and CNVs of these two groups to compare their genomic alteration patterns for any potential difference. The top mutated genes in EBV tumors were *ARID1A* (46.8%) and *PIK3CA* (42.6%), consistent with previous report.^7^ In the H. pylori group, mutations occurred most frequently in *TP53* (47%) and *CDH1* (12%). To compare recurrent mutations, only genes mutated in greater than or equal to 10 samples were included. *PIK3CA*, *ARID1A*, and *PTEN* mutations were significantly enriched in EBV subtype while *TP53* mutation were significantly enriched in H. pylori subtype (Figure 4A). After multiple testing adjustment by FDR, enrichment within *PIK3CA*, *ARID1A*, and *TP53* remained significant (Table S1). Among these three genes, missense was the most common type of variant detected (49%), followed by stop gain (19%), and frameshift variants (19%) (Table 2). In terms of the mutation rate in these three genes, the majority of patients (91%) had only one variant per gene, with 9% having two variants per gene, and one patient having four variants per gene. According to ANNOVAR^16^ annotations, half of the variants detected in these genes were classified as either pathogenic or likely pathogenic (pathogenic/likely pathogenic (30%), likely pathogenic (12%), pathogenic (8%) (Table 3). In addition, 38% and 40% of variants in these genes were predicted to be damaging (D) by SIFT^17^ and PolyPhen,^18^ respectively (see Data S1 for complete data). Across all patients and genes, missense was also the most common type of mutation detected (65%), followed by frameshift (14%) and stop gain (7%) variants (see Data S2 for complete data). Copy number gain in *MCL1* (14.5%), *TERC* (9%), and *CCNE1* (8.5%) and copy number loss in *PTPRD* (8.5%) were the most common CNVs in H. pylori subtype. On the contrary, CNVs were less common in EBV‐positive tumors with the top ones being *MCL1* gain (8.5%) and *ZNF217* gain (8.5%). Among genes that had altered copy numbers in more than 10 samples, only the frequency of *CCNE1* gain was significantly different between H. pylori and EBV subtypes (Figure 4B, Table S1).

**TABLE 2:** Distribution of top five mutation types for genes (ARID1A, PIK3CA, and TP53) found to significantly differ between H. pylori and EBV subtypes after multiple test correction

Mutation type	Mutation count	Percentage
Missense	94	49.2%
Stop gain	37	19.4%
Frameshift	36	18.8%
Splice donor	7	3.7%
Inframe deletion	5	2.6%

Minor mutation types were omitted, these include splice acceptor, inframe insertions, splice region, and protein‐altering variants.

**TABLE 3:** Distribution of ANNOVAR clinical significance annotations for mutations in genes (ARID1A, PIK3CA, and TP53) found to significantly differ between H. pylori and EBV subtypes

Mutation pathogenicity	Mutation count	Percentage
Unknown	83	43.5%
Pathogenic/ Likely pathogenic	57	29.8%
Likely pathogenic	22	11.5%
Pathogenic	15	7.9%
Conflicting interpretations	11	5.8%
Likely_benign	1	0.5%
Other	1	0.5%
Uncertain_significance	1	0.5%

Lastly, because H. pylori‐positive samples tend to have a low CIS score (85.5%, *p* < 0.001), we also compared H. pylori‐positive subtype and GS subtype. Top mutated genes in GS tumors were *TP53* (55%), *CDH1* (17.6%), *ARID1A* (16%), *PIK3CA* (8.3%), and *RHOA* (6.6%). Notably, *RHOA* and *CDH1* were also frequently mutated in GS tumors in the TCGA study.^7^ Mutations in *ARID1A*, *POLE*, *SETD2*, *TP53*, and *CDKN2A* were enriched in GS samples while mutations in *AKT3*, *EPAS1*, *BTK*, and *MLH1* were enriched in H. pylori‐positive samples (Figure 4C, Table S2). Copy number gain of *NFE2L2*, *TERC*, *MCL1*, and *TOP1* were enriched in H. pylori‐positive samples (Figure 4D, Table S2). No copy number loss events were found to be enriched in either group.

**FIGURE 5:**
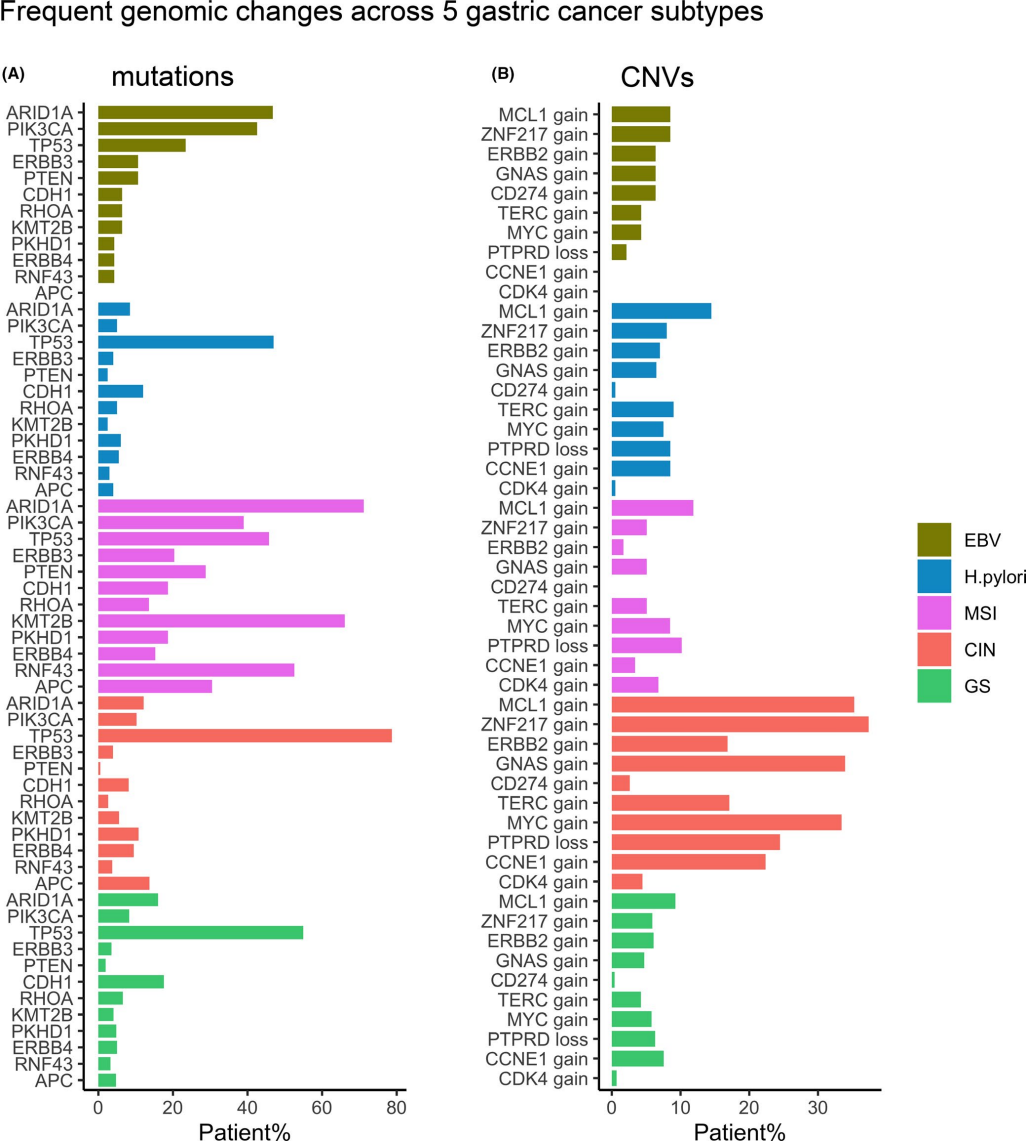
Frequent mutations (A) and CNV (B) across five gastric cancer subtypes

Genes exhibiting the most frequent mutations or CNV events among all subtypes were summarized in Figure 5A and B. Consistently, MSI subtype had higher mutation frequency in the genes listed than any other group due to mismatch repair deficiency. Interestingly, despite the relatively low mutation rate of other genes in CIN subtype, *TP53* was mutated in 79% of all samples. This is consistent with the high alteration frequency of *TP53* (71%) observed in CIN tumors from the TCGA.^7^ As a major tumor suppressor, *P53* controls genome stability and its dysfunction could induce large scale genomic alteration seen in the CIN subtype.^21^ Also, CIN subtype showed a higher copy number alteration frequency of listed genes than other subtypes. However, *CD274* gain occurred at a moderate frequency only in EBV tumors only (6.38%), but infrequently in other subtypes.

## 4 DISCUSSION

In this study, we uncovered the genomic landscape and tumor microbial profile of H. pylori‐positive gastric tumor and compared it with previously characterized subtypes.^7^ Using a targeted hybrid‐capture panel, we examined various genomic features of the tumor including mutation, mutational signature, TMB, MSI, and CNV. Identification of DNA reads from infectious agents in the tumor samples indicates that the deep sequencing platform has promising utility in uncovering the characteristics of tumor microbial environment. While EBV and H. pylori infections are often observed in gastric cancer, co‐infection with both H. pylori and EBV occurred at a low frequency in this cohort.

Using off‐target reads from targeted sequencing for detection of EBV or H. pylori has certain limitation on our definition of EBV and H. pylori status. The presence of EBV or H. pylori means only tumors with high levels of viral or bacterial reads are detected, and those tumors are classified here as EBV or H. pylori‐positive. Thus, this study only focused on the comparison between two groups with high or low EBV or H. pylori levels in their tumor tissues.

Like EBV‐positive tumors, H. pylori‐positive tumors had a lower CIS score compared to other subtypes. TMB was similar among non‐MSI subtypes, but H. pylori‐positive gastric tumors had a lower MSI score. Consistent with a previous report, H. pylori infection is associated with double‐strand break and reduced DNA repair efficiency in gastric cancer,^22^ which is evident from the mutational signatures seen in H. pylori‐positive tumor. Furthermore, we also found APOBEC‐associated mutational signature in this subtype, which could be caused by H. pylori‐induced mutagenesis.^23^ Overall, H. pylori‐positive gastric cancer appears to have a relatively stable genome. Nonetheless, H. pylori‐positive subtypes harbor some unique gene mutations and CNVs compared to EBV and GS subtypes. Specifically, H. pylori‐positive tumors showed depletion of PIK3CA and ARID1A mutations, and enrichment of TP53 mutations. Many of these mutations were predicted to impact protein function, and were predominantly pathogenic or likely pathogenic. These findings suggest that the differences seen between H. pylori and EBV subtypes hold biological and clinical importance for better understanding and treatment of gastric cancer. Future studies examining the mechanistic details of H. pylori‐associated genomic change could further our understandings of this unique subtype of gastric cancer. Additionally, whether these subtypes have different response to standard therapy or immunotherapy is of great clinical interest.

## CONFLICT OF INTEREST

HB, AW, MH, and XW are employees and/or shareholders of Nanjing Geneseeq Technology, Inc. All other authors declared no conflicts of interest.

## ETHICS APPROVAL AND PATIENT CONSENT

Study conforms to the Declaration of Helsinki. Written informed consent was collected from each patient upon sample collection according to the protocols approved by the ethical committee of their respective hospitals.

## Supporting information

Table S1‐S2

Supplementary Material

Supplementary Material

Supplementary Material

Supplementary Material

## Data Availability

The data sets used and/or analyzed during the current study are available from the corresponding author on reasonable request. Certain restrictions may apply.
