# Supplementary material for: Distinct genomic profile in h. pylori‐associated gastric cancer
Source: Cancer Med. 2021 Mar 9;10(7):2461–9. doi: 10.1002/cam4.3765 (PMC7982637; doi:10.1002/cam4.3765)
Supplement: Supplementary file 1 — Table S1‐S2 [file CAM4-10-2461-s005.docx]

**Supplementary Tables**

**Table S1**. Significant genomic alteration differences between H. pylori and EBV subtypes.

| **Mutational difference** | |  |  |  |  |  |  |  |
| --- | --- | --- | --- | --- | --- | --- | --- | --- |
| gene | n.heli | n.ebv | p.value | p.adj | heli_ratio | ebv_ratio | fold_change | direction |
| PIK3CA | 10 | 20 | 6.47E-10 | 7.11E-09 | 0.05 | 0.4255 | 0.1175 | decrease |
| ARID1A | 17 | 22 | 7.08E-09 | 3.89E-08 | 0.085 | 0.4681 | 0.1816 | decrease |
| PTEN | 5 | 5 | 0.02413 | 0.06636 | 0.025 | 0.1064 | 0.235 | decrease |
| TP53 | 94 | 11 | 0.003171 | 0.01163 | 0.47 | 0.234 | 2.009 | increase |
| **CNV difference** | |  |  |  |  |  |  |  |
| gene | n.heli | n.ebv | p.value | p.adj | heli_ratio | ebv_ratio | fold_change | direction |
| CCNE1 | 17 | 0 | 0.04877 | 0.4389 | 0.085 | 0 | Inf | increase |

Note: n.heli is number of subjects in H. pylori subtype group with mutations or CNV in gene. n.ebv is number of subjects in EBV subtype group with mutations or CNV in gene. p.adj is p-value after FDR adjustment. heli_ratio is n.heli / total number of subjects in H.pylori subtype group. ebv_ratio is n.heli / total number of subjects in EBV subtype group. fold_change = heli_ratio/ebv_ratio. Direction: if fold_change is > 1, increase; if fold_change < 1, decrease.

**Table S2**. Significant genomic alteration differences between H. pylori and GS subtypes.

| **Mutational difference** | |  |  |  |  |  |  |  |
| --- | --- | --- | --- | --- | --- | --- | --- | --- |
| gene | n.heli | n.gs | p.value | p.adj | heli_ratio | gs_ratio | fold_change | direction |
| ARID1A | 17 | 163 | 0.00616 | 0.4743 | 0.085 | 0.1603 | 0.5303 | decrease |
| POLE | 0 | 23 | 0.02271 | 0.6995 | 0 | 0.02262 | 0 | decrease |
| TP53 | 94 | 559 | 0.04365 | 0.7352 | 0.47 | 0.5497 | 0.855 | decrease |
| SETD2 | 1 | 29 | 0.04687 | 0.7352 | 0.005 | 0.02852 | 0.1753 | decrease |
| CDKN2A | 2 | 38 | 0.0495 | 0.7352 | 0.01 | 0.03736 | 0.2677 | decrease |
| AKT3 | 7 | 6 | 0.002072 | 0.3191 | 0.035 | 0.0059 | 5.932 | increase |
| EPAS1 | 5 | 5 | 0.01418 | 0.6995 | 0.025 | 0.004916 | 5.085 | increase |
| MLH1 | 5 | 6 | 0.02259 | 0.6995 | 0.025 | 0.0059 | 4.237 | increase |
| BTK | 6 | 10 | 0.03452 | 0.7352 | 0.03 | 0.009833 | 3.051 | increase |
| **CNV difference** | |  |  |  |  |  |  |  |
| gene | n.heli | n.gs | p.value | p.adj | heli_ratio | gs_ratio | fold_change | direction |
| NFE2L2 | 7 | 6 | 0.002072 | 0.0601 | 0.035 | 0.0059 | 5.933 | increase |
| TERC | 18 | 43 | 0.007557 | 0.1096 | 0.09 | 0.04228 | 2.129 | increase |
| MCL1 | 29 | 94 | 0.02897 | 0.2406 | 0.145 | 0.09243 | 1.569 | increase |
| TOP1 | 11 | 24 | 0.03318 | 0.2406 | 0.055 | 0.0236 | 2.331 | increase |

Note: n.heli is number of subjects in H. pylori subtype group with mutations or CNV in gene. n.gs is number of subjects in GS subtype group with mutations or CNV in gene. p.adj is p-value after FDR adjustment. heli_ratio is n.heli / total number of subjects in H.pylori subtype group. gs_ratio is n.heli / total number of subjects in GS subtype group. fold_change = heli_ratio/gs_ratio. Direction: if fold_change is > 1, increase; if fold_change < 1, decrease.
